# Shifting Baselines in Antarctic Ecosystems; Ecophysiological Response to Warming in *Lissarca miliaris* at Signy Island, Antarctica

**DOI:** 10.1371/journal.pone.0053477

**Published:** 2012-12-28

**Authors:** Adam J. Reed, Sven Thatje, Katrin Linse

**Affiliations:** 1 Ocean and Earth Science, University of Southampton, National Oceanography Centre Southampton, Southampton, United Kingdom; 2 British Antarctic Survey, Natural Environment Research Council, Cambridge, United Kingdom; University of Thessaly, Greece

## Abstract

The Antarctic Peninsula has experienced a rapid increase in atmospheric temperature over the last 50 years. Whether or not marine organisms thriving in this cold stenothermal environment are able to cope with warming is of concern. Here, we present changes to the growth and shell characteristics of the ecologically important, small and short lived brooding bivalve *Lissarca miliaris* from Signy Island, Antarctica. Using material collected from the 1970's to the present day, we show an increase in growth rate and adult shell deterioration accompanied by a decrease in offspring size, associated with an increase in annual average temperatures. Critical changes to the bivalve's ecology seen today evidence the problem of a shift in baseline since the onset of warming recorded in Antarctica. These small bivalves are demonstrating ecophysiological responses to subtle warming that, provided warming continues, could soon surpass a physiological tipping point, adding to warming associated threats such as increased predatory pressure and ocean acidification.

## Introduction

The rate of atmospheric warming at the Antarctic Peninsula has been around 0.56°C decade^−1^ since 1950 [1], higher than the global average and most extreme in winter with an increase of 5–6°C over the past 50 years [2]. Observed glacial retreat [3], reduced sea ice formation [4] and a regional increase of 1°C in the upper ocean layer in summer [5], have all been attributed to this temperature increase. Ecosystem responses can be difficult to identify but to date include changes in plankton biomass [6], penguin distribution and krill abundances [7]. Investigating physiological responses to thermal stress may also be important in understanding ecosystem changes occurring at population levels [8], [9].

A problem facing ecologists when studying ecosystem change is finding a baseline in which to measure change against. The term ‘shifting baselines’ was first used to describe fishery scientists who failed to use historic data to evaluate the status of the ecosystem, instead using the ecosystem status at the start of their career as the baseline for change [10], [11]. This concept has since been discussed to include a wide range of ecosystems that are only studied whilst in decline; in rare cases no historic data being available to develop a suitable baseline [12]. This is certainly the situation for many coral reefs [13], [14], benthic environments [15] and in rocky shore ecology [16], where often no adequate baselines are currently attainable.

Long-lived marine bivalves are often used to study faunal response to environmental change as different variables may be recorded by growth increments and shell chemistry. A commonly studied species is the sub-Arctic bivalve *Arctica islandica* that can live in excess of 350 years [17], while in the Antarctic, 40 year old *Laternula elliptica* shells have been modelled to infer details into past growth rate and production [18]. While providing an invaluable insight into responses to climatic variability, the slow growth rates of long-lived species may hide inter-decadal variation. Interpretation of results can also be difficult in a macro-ecological context when communities are dominated by comparatively small, short lived and faster growing species. Southern Ocean bivalves are typically thin shelled [19] with calcium difficult to extract from seawater at low temperatures [20], making them potentially vulnerable to changes in temperature, decreasing CaCO^3^ saturation [21], [22] and predation from invasive durophagous predators in the course of warming [23], [24].

The philobryid bivalve *Lissarca miliaris* (Philippi 1845) is a small species (typically <5 mm) commonly found in the inter- and sub-tidal regions attached to macro-algae by byssal threads around Signy Island, Antarctica. They are relatively short lived, living up to 7 years, and brood a maximum of 70 young for 18 months [25], [26]. Populations occur along the Antarctic Peninsula, Scotia Sea, and sub-Antarctic, often in dense aggregations. Within Borge Bay, Signy Island, *L. miliaris* are the most dominant species of mollusc both by weight and number living on the abundant macroalgae *Desmarestia anceps* [25]. As an ecologically important species with a wide distribution and comparatively short life-span, *L. miliaris* make a good model species for identifying changes in the Antarctic environment. Using published data from 1972 [25], specimens collected in 1976, 2002, 2011 and 2012, we study the effects of regional temperature increase on the growth rates and shell characteristics of *L. miliaris*. This study highlights the importance of historic data in polar areas, the striking response of shelled invertebrates to subtle changes in temperature, and the risk of shifting baselines affecting our perception of the ‘pristine’ Antarctic ecosystem.

## Materials and Methods

### Ethics Statement

Collections were not made from any protected or private sites within Antarctica. This study did not involve endangered or protected species. All necessary permits were obtained for the described field collections, within the Antarctic Act (1994).

### Collection

**Figure 1:**
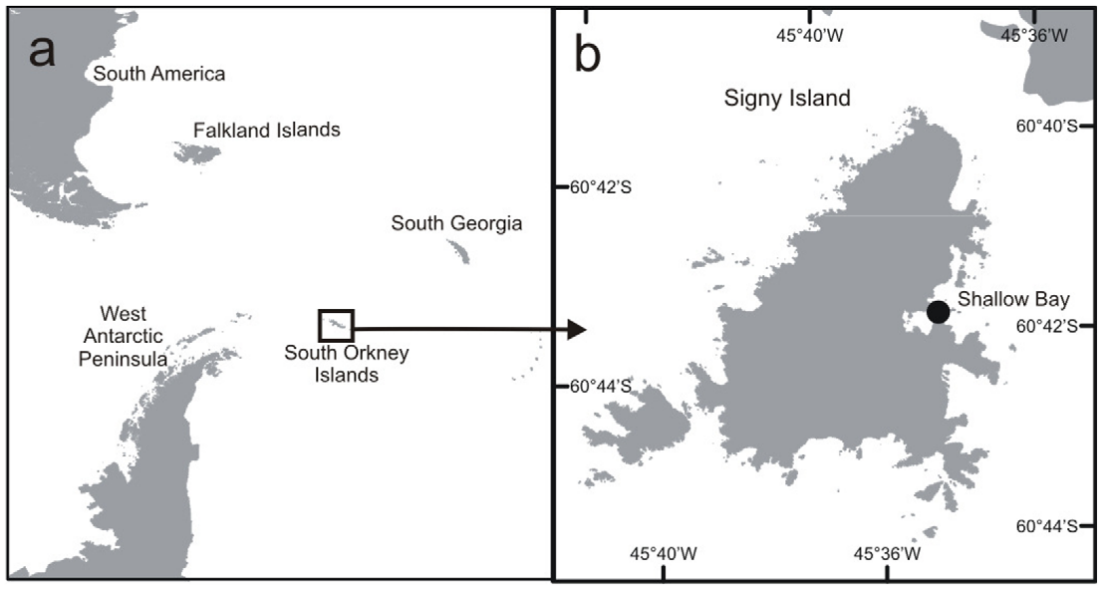
Map of study area a) Antarctic Peninsula showing location of South Orkney Islands; b) Signy Island (62°42′S, 45°36′W) showing location of the intertidal Shallow Bay within Borge Bay.

We analysed a total of 808 hand-collected intertidal *Lissarca miliaris* near the British Signy Base at Shallow Bay, within Borge Bay, Signy Island (60°42′S, 45°36′W; Figure 1). This was made up of 226 specimens fixed in formalin but stored in ethanol from 1976, 462 specimens from April 2002 in 96% ethanol, 68 specimens in February and March 2011 fixed in 96% ethanol and 52 specimens in March 2012, 10 dried at 30°C, 42 fixed in 96% ethanol. The specimens from 2002 were collected by hand as part of the R/V *Polarstern* LAMPOS (ANT XIX/5) expedition [27].

### Growth

Each specimen of *L. miliaris* was measured along the maximum distance across the shell using a stereo-microscope (precision ±0.025 mm). Annual growth measurements were counted by eye and assumed to be annual [25], [28]. Size-at-age data were analysed using the von-Bertalanffy growth function (VBGF) [29]:
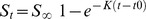
where *S_t_* is length, *S_∞_* is asymptotic length, *K* is growth coefficient, *t* is age and *t_0_* is age when size equals zero.

Overall growth performance (P) was computed using *K* and *S_∞_* derived from the VBGF equation;
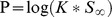



### Prodissoconch sizes

Prodissoconch sizes were measured by image analysis of micrographs taken with camera mounted stereo-microscope. Only ‘0 year’ and ‘1 year’ specimens with an undamaged prodissoconch were used for this analysis. 2011 and 2012 data were pooled as sampling of these later specimens included low numbers of ‘0 year’ animals. A total of 84 measurements were made (1976 n = 26, 2002 n = 47, 2011/2012 n = 11).

### Shell analysis

The right valve from three specimens in each collection with 5 growth rings was used for shell analysis. Shells were embedded into Epoxy resin and were cut with a 100×0.37 mm diamond low speed saw along the longest growing margin from the umbo. Cut blocks were polished with graded diamond-coated sanding cloths to 1 µm and carbon coated before Scanning Electron Microscopy (SEM) analysis.

Energy dispersive spectrometry (EDS) was used to provide qualitative and quantitative measurements of the dominant trace elements in the shell structures. EDS analysis was taken through the shell at transects 600 µm apart from the umbo, representing average growth per year to ensure all years of growth were accounted for. The middle layer showed least variability in shell chemistry (unpublished data) and was used to generate quantitative measurements. Sections were analysed with a Leo 1450 scanning electron microscope with a PGT microanalysis energy dispersive system. Ratios of trace elements with calcium were used to identify changes in chemistry over time.

## Results

**Figure 2:**
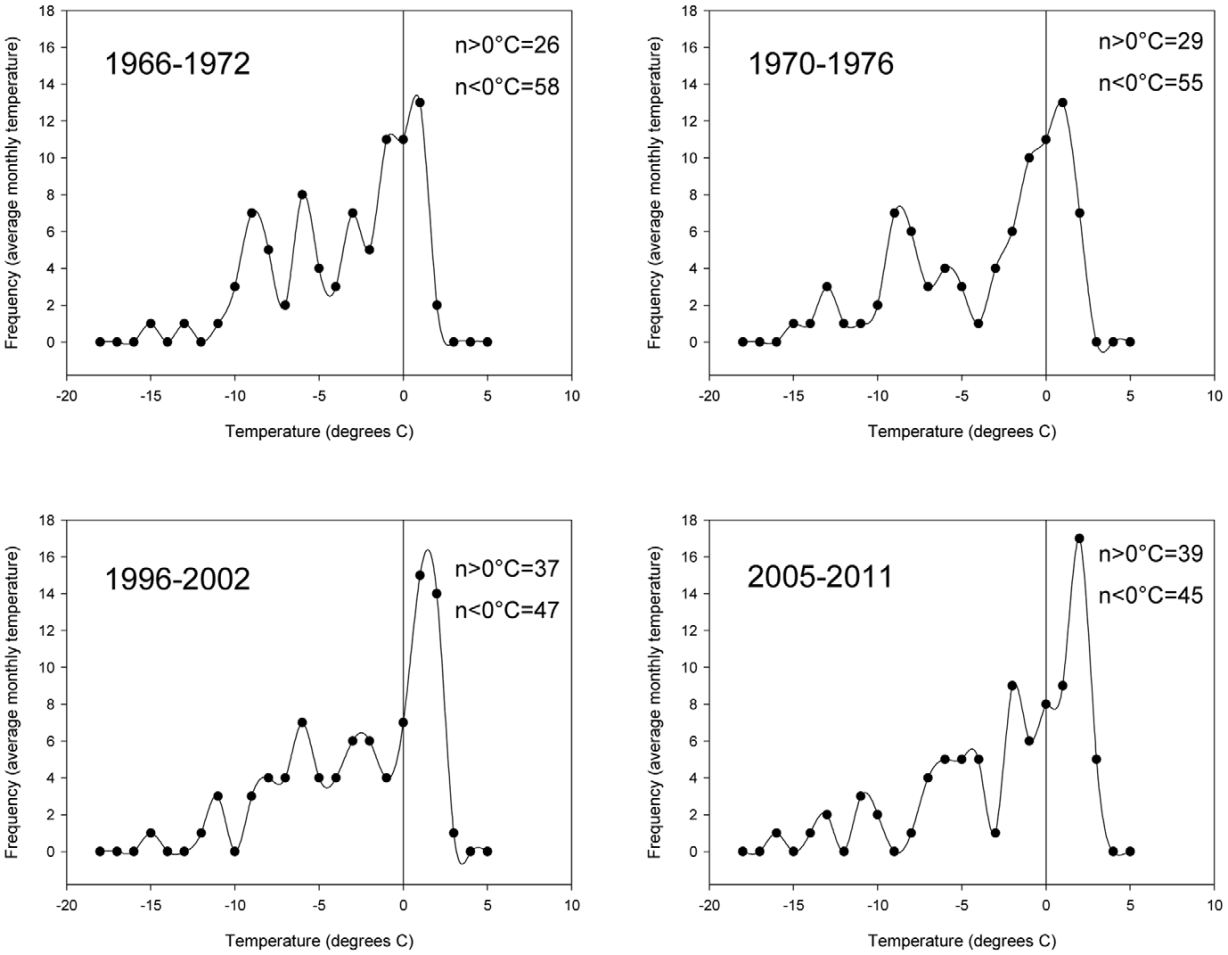
Frequency of mean monthly temperatures at Orcadas Research Station, Laurie Island for the 7 years up to the specimen collection dates, demonstrating the number of months averaging below and above 0°C over the life of the adult *Lissarca miliaris*. Vertical line represents 0°C.

**Figure 3:**
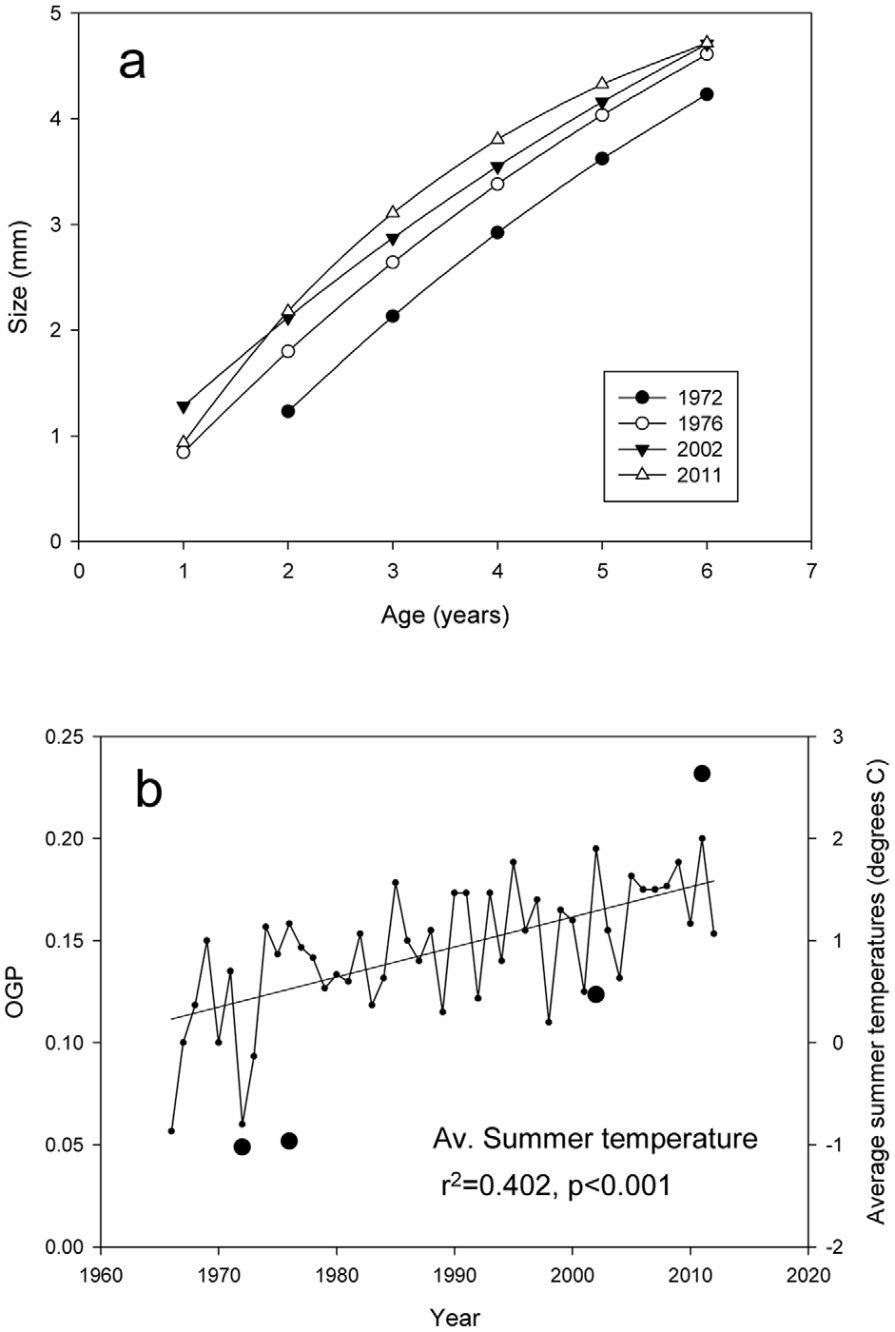
Growth parameters of *Lissarca miliaris* from Signy Island. a) von Bertalanffy growth function from size-at-age data of *L. miliaris* from 1972–2011; b) Overall growth performance of *L. miliaris* from 1972–2011 (large circles) displaying average summer air temperatures from 1966 to 2012 (small circles).

Using monthly temperature date from the Argentinean Orcadas research station, an increase in air temperature is observed since the collections of *L. miliaris* began. The increase in air temperature for our collections is described by the number of months a year the average temperature was above 0°C, over the seven-year life span of the specimens collected (Figure 2). Months above 0°C increased from 26 months between 1966 and 1972 to 39 months between 2005 and 2011. Peak frequency of months above 0°C shifted from 1°C to 2°C over the same period. Average summer temperatures between 1970 and 2011 show high inter-annual variability (Figure 3b) but a significant increase in air temperature (r^2^ = 0.402, p<0.001).

The calculated growth constant *K*, representing ‘rate of growth’, increases from 0.130 in 1972 to 0.208 in 2002 and 0.290 in 2011 while the asymptotic maximum size (*S_∞_*) decreases from 8.61 mm in 1972 to 5.88 mm in 2011 (Figure 3a, Table S1). The overall growth performance (OGP) for each sample shows a five-fold increase from the 1970's samples to 2011 (Figure 3b). Maximum prodissoconch lengths (P1) of *L. miliaris* have decreased from 714.5 µm±5.17 in 1976 to 694.3 µm±7.14 in 2011 and 2012 while the maximum height have increased slightly from 449.0 µm±1.60 to 458.9 µm±1.52 (Table 1). No P1 data were available for the collection in 1972. The resulting decrease in length/height ratio from 1.598±0.017 to 1.516±0.029, was significant (Kruskal-Wallis H = 10.10, df = 2, p<0.01).

**Table 1:** Maximum prodissoconch length, height and ratio including standard error for *Lissarca miliaris* collected at Shallow Bay, Signy Island in 1976, 2002 and 2011/12.

Year	Length (µm)	Standard Error	Height (µm)	Standard Error	Length/height ratio	n
1976	**714.5**	5.17	**449.0**	6.31	1.60	26
2002	**699.2**	4.96	**458.0**	3.79	1.53	46
2011&2012	**694.3**	7.14	**458.9**	3.63	1.52	11

The difference in length/height ratio between the collections was significant (Kruskal-Wallace H = 10.10, df = 2, P<0.01).

**Figure 4:**
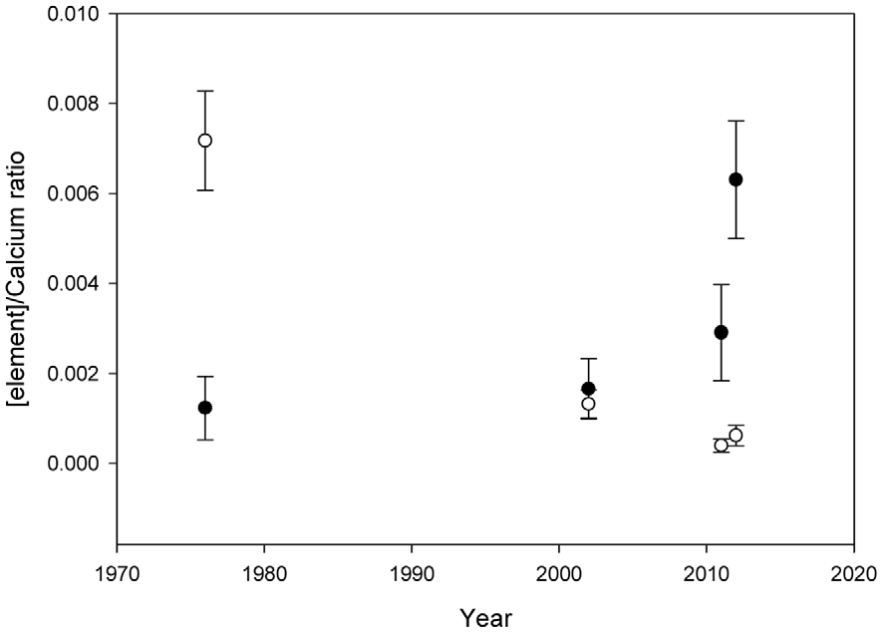
Element/Calcium ratios (mean ± standard error) of *Lissarca miliaris* shells from Signy Island. Strontium/Calcium ratios (filled circle) from 1976–2012 and Phosphorus/Calcium ratio (open circle) from 1976–2012. Error bars represent standard error.

**Figure 5:**
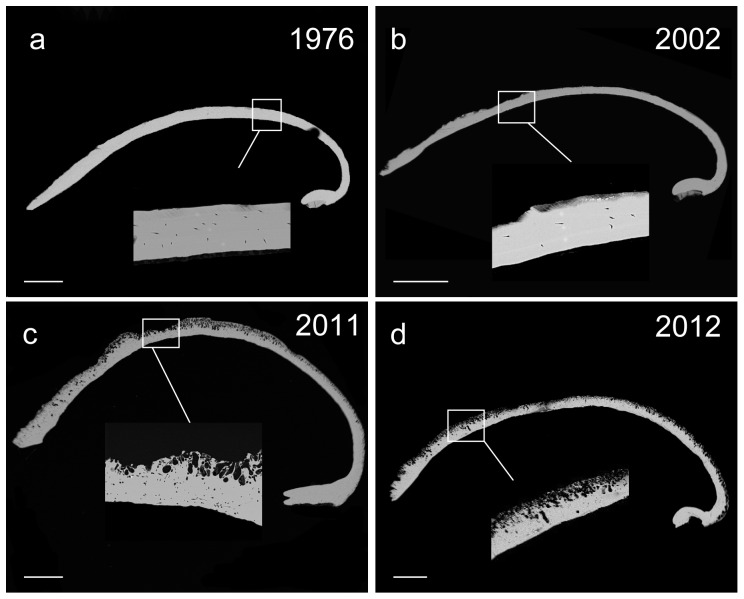
SEM backscatter images of section adult *Lissarca miliaris* shells with higher magnification inlays. a) 1976 section; b) 2002 section; c) 2011 section; d) 2012 section. Scale bars = 500 µm.

Shell formation and chemistry have also changed over time. The Strontium∶Calcium ratio increased from 0.0012±0.0007 in 1976 to 0.0029±0.0011 in 2011 (Figure 4). Strontium in 2002 was similar to 1976 (0.0017±0.0007) while the dried material from 2012 was highest (0.0063±0.0013). The Phosphorus∶Calcium ratio was very low but decreased from 0.0013±0.0003 to 0.0006±0.0002 between 1976 and 2012. Maximum shell thickness increased from 166 µm in 1976 to 276 µm in 2011 and 206 µm in 2012 and is associated with deteriorating shell quality and increased shell repair (Figure 5). The integrity of the shells from 2011 and 2012 are compromised by endolithic decay causing erosion of the upper layer of shell and the subsequent secondary shell deposition results in a thicker shell (Figure 5c&d). Decay is not observed in the 1976 collection (Figure 5a), and cannot be confidently identified in the 2002 collection (Figure 5b). To confirm dissolution was not an artefact of preservation, the 2012 specimens were dried without contact with formalin/ethanol, and only the 1976 material had been fixed in formalin (subsequently ethanol stored).

## Discussion

The South Orkney Islands have one of the longest time series of recorded air temperature, dating back to 1903 at the Argentinean research station. Temperatures recorded fit closely to the monthly air temperature data collected at the British Signy research station from 1945 until 1995 when data collection halted (Figure S1) and have shown an average increase of 0.20°C decade^−1^ over 100 years [1], although much of this changes appears to be in the last 50 years. The increased growth rate of *L. miliaris* over the past 40 years is likely to be a response to this changing temperature in the region. Although no record of sea-water temperature is available for this period at Shallow Bay, the intertidal distribution of *L. miliaris* would make an increase in air temperature a significant factor affecting its physiology. Growth rate is closely linked with two factors, food availability and temperature [18], [30]. Chlorophyll *a* values in Borge Bay show high levels of interannual variability and are considered to be high in the summer while extremely low in the winter [31], [32]. The duration of these blooms might be expected to increase with air temperature as nutrients are rarely limiting, although no evidence of this is observed in the water sampling programmes of 1972–1994 [31], [32].

*L. miliaris* has a wide distribution and is commonly found as far north as South Georgia [33]. Air temperatures experienced at Signy Island still remain cooler than the temperatures experienced by other populations in the sub-Antarctic and assuming connectivity between populations, the increase in temperature at Signy Island is unlikely to have reached the species thermal limit. OGP provides a method to compare ‘how well’ an organism grows [34], [35] and a graphical representation of growth, a higher OGP suggesting a less stressful environment in which to grow. The five-fold increase in OGP over 40 years is in contrast to the larger, infaunal bivalve *Laternula elliptica* from King George Island [18] which showed a decrease in OGP over 40 years, inferred from shell growth per year in old specimens. However, the two species differ in their distribution, with *L. miliaris* perhaps favouring the conditions that regional environmental changes have brought.

The most striking change between collections was the condition, and composition of the shells. Strontium in aragonite structures is very well studied and its relationship with temperature is used as a climatic proxy [36], [37]. During faster crystal growth rates, near-surface migration of ions, which expel impurities, are less efficient and an increase in strontium replacement of calcium ions might be expected [38]. Strontium has also been found to be correlated with temperature indirectly by increased growth rates in marine gastropods [39]. The increased ratios of strontium in the shells of *L. miliaris* are therefore likely to be linked to the increased growth rates observed, although other abiotic influences may also affect this relationship. Phosphorus is incorporated from the surrounding seawater, suggesting a change in dissolved phosphorus levels.

The shell damage observed in 2011 and 2012 shells resembles the decay caused by photosynthetic bacteria [40], [41]. Shell dissolution in the Antarctic is very poorly understood [22] and only one study has described endolithic algae on the shells of the bivalve *Adamussium colbecki* [42], and at Signy Island a single example of macro algae causing shell erosion in the limpet *Nacella concinna* [43]. The dissolution observed appears to be greater on older specimens where the protective periostracum has become eroded; algal cells are observed on the freshly preserved adult 2012 specimens (Figure S2). All *L. miliaris* shells in this study were of the same age, but the degree of shell dissolution was much greater in the 2011/2012 specimens compared to the almost perfect shell surface in 1976 specimens. This is also reflected in the thickening of shells as the bivalves attempt to repair their damaged shells with secondary calcification (Figure 5c). The temperature increase in the region may be encouraging faster growth of such endolithic bacteria and the warmer winters experienced over the past decade may be increasing the survival of such organisms during the light and temperature limiting months.

Shell repair is energetically costly and requires an energy reallocation, potentially negatively affecting reproductive output [44], [45] and the decrease in Prodissoconch 1 sizes is indicative of this decrease in reproductive effort per egg. Secreted by the bivalve in early development, the prodissoconch size can be directly linked to egg size and differences in egg size as small as 10 µm can be reflected in significant differences in prodissoconch 1 length [46]. Smaller egg sizes in *L. miliaris* might suggest less energy available for development and a higher risk of larval mortality. The observation of thickened shells and reduced reproductive output demonstrates the energetic trade-off involved in maintaining shell integrity. Some brooding bivalves are known to decrease immune responses leaving a greater susceptibility to parasite infection [47]. Many Antarctic invertebrates brood for over a year; if *L. miliaris* have suppressed immunity for 18 months of brooding, their reduced resistance to endolithic parasites and cost of shell repair may impact their survival.

*L. miliaris* at Signy Island show the vulnerability of Antarctic fauna by rapidly responding to only a subtle change in temperature over four decades, demonstrating the importance of having a realistic baseline for measuring change. The growth rate response already occurring is startling but before a physiological tipping point is reached other factors, including the shell dissolution described in this study, may affect future growth and survival. We highlight the need to investigate other small, shelled organisms that may already be in a highly altered ecological state in polar ecosystems, although only by using historic and archived material can the true extent of environmental change be measured. It is likely too late for realistic baselines to be established for measuring such change and any management approach must therefore be assessed against an already altered ecosystem state. Antarctic ecosystems are often perceived to be pristine [48] but this study demonstrates changes that have been occurring unnoticed for at least 40 years, and other critical changes could be occurring in ecosystems that are assumed to be stable. The immediate concern for *L. miliaris* and the polar shelled organisms it represents is the energetic cost of shell repair affecting reproduction, but also the reduced resilience to the inevitable pressures associated with invasive species, ocean acidification and predictions of further warming of the Southern Ocean [24] that by far exceed the comparatively subtle warming seen to date.

## Supporting Information

Figure S1 **Mean annual temperature from 1947–1995 from the British Signy Island base (dotted line) and Argentine Orcadas, Laurie Island Base (solid line).** Signy Island temperature data collection stopped in 1995.
(PDF)

Figure S2 **Adult** ***Lissarca miliaris*** **from Shallow Bay, Signy Island collected in 2012.** Endolithic algae can still be seen in green, covering the shells shortly after fixation. Scale bars = 2 mm.
(PDF)

Table S1 **Parameters of the von Bertalanffy growth function and calculated overall growth performance (OGP) for** ***Lissarca miliaris*** **at Shallow Bay, Signy Island.**
(PDF)

## References

[1] Turner J, Colwell SR, Marshall GJ, Lachlan-Cope TA, Carleton AM, et al (2005) Antarctic climate change during the last 50 years. Int J Climatol 25: 279–294.

[2] Vaughan DG, Marshall GJ, Connolley WM, Parkinson C, Mulvaney R, et al (2003) Recent rapid regional climate warming on the Antarctic Peninsula. Clim Change 60: 243–274.

[3] Cook AJ, Fox AJ, Vaughan DG, Ferrigno JG (2005) Retreating glacier fronts on the Antarctic Peninsula over the past half-century. Science 308: 541–544. 15845851 10.1126/science.1104235

[4] Stammerjohn SZ, Martinson DG, Smith DG, Lannuzzi RA (2008) Sea Ice in the western Antarctic Peninsula region: spatio-temporal variability from the ecological and climate change perspectives. Deep-Sea Res Part II 55: 2041–2058.

[5] Meredith MP, King JC (2004) Rapid climate change in the ocean west of the Antarctic Peninsula during the second half of the 20th century. Geophys Res Lett 32: L19604 doi:10.1029/2005GL024042.

[6] Schofield O, Ducklow HW, Martinson DG, Meredith MP, Moline MA, et al (2010) How do polar marine ecosystems respond to rapid climate change? Science 328: 1520–1523. 20558708 10.1126/science.1185779

[7] Fraser WR, Hoffmann EE (2003) A predator's perspective on causal links between climate change, physical forcing and ecosystem response. Mar Ecol Prog Ser 265: 1–15.

[8] Pörtner HO (2001) Climate change and temperature dependant biogeography: oxygen limitation of thermal tolerance in animals. Naturwissenschaften 88: 137–146. 11480701 10.1007/s001140100216

[9] Pörtner HO (2010) Oxygen- and capacity-limitation of thermal tolerance; a matrix for integrating climate-related stressor effects in marine ecosystems. J Exp Biol 213: 881–893. 20190113 10.1242/jeb.037523

[10] Pauly D (1995) Anecdotes and the shifting baseline syndrome of fisheries. Trends Ecol Evol 10: 430. 21237093 10.1016/s0169-5347(00)89171-5

[11] Pinnegar JK, Engelhard GH (2008) The ‘shifting baseline’ phenomenon: a global perspective. Rev Fish Biol Fisher 18: 1–16.

[12] Jackson JBC, Kirby MX, Berger WH, Bjorndal KA, Botsford LW, et al (2001) Historical overfishing and the recent collapse of coastal ecosystems. Science 293: 629. 11474098 10.1126/science.1059199

[13] Cramer KL, Jackson JBC, Angioletti CV, Leonard-Pingel J, Guilderson TP (2012) Anthropogenic mortality on coral reefs in Caribbean Panama predates coral disease and bleaching. Ecol Lett 15: 561–567. 22462739 10.1111/j.1461-0248.2012.01768.x

[14] Knowlton N, Jackson JBC (2008) Shifting baselines, local impacts and global change on coral reefs. PLoS Biol 6: e54. 18303956 10.1371/journal.pbio.0060054 PMC2253644

[15] Villnäs A, Norkko A (2011) Benthic gradients and shifting baselines: implications for assessing environmental status. Ecol Appl 21: 2172–2186. 21939052 10.1890/10-1473.1

[16] Roy K, Collins AG, Becker BJ, Begovic E, Engle JM (2003) Anthropogenic impacts and historical decline in body size of rocky intertidal gastropods in southern California. Ecol Lett 6: 205–211.

[17] Schöne BR, Fiebig J, Pfeiffer M, Gleβ R, Hickson J, et al (2005) Climate records from a bivalved Methuselah (*Arctica islandica*, Mollusca; Iceland). Palaeogeogr Palaeoclimatol Palaeoecol 228: 130–148.

[18] Brey T, Voigt M, Jenkins K, Ahn I-J (2011) The bivalve *Laternula elliptica* at King George Island – A biological recorder of climate forcing in the West Antarctic Peninsula region. J Mar Syst 88: 542–552.

[19] Nicol D (1967) Some characteristics of cold-water marine pelecypods. J Palaeo 41: 1330–1340.

[20] Harper EM (2000) Are calcitic layers an effective adaptation against shell dissolution in the Bivalvia? J Zool 251: 179–186.

[21] Fabry VJ, McClintock JB, Mathis JT, Grebmeir JM (2009) Ocean acidification at high latitudes: The Bellwether. Oceanogr 22: 160–171.

[22] McClintock JB, Angus RA, McDonald MR, Amsler CD, Catledge SA, et al (2009) Rapid dissolution of shells of weakly calcified Antarctic benthic macroorganisms indicates high vulnerability to ocean acidification. Antarct Sci 21: 449–456.

[23] Aronson RB, Thatje S, Clarke A, Peck LS, Blake DB, et al (2007) Climate change and invasibility of the Antarctic benthos. Annu Rev Ecol Evol Syst 38: 129–154.

[24] Aronson RB, Thatje S, McClintock JB, Hughes KA (2011) Anthropogenic impacts of marine ecosystems in Antarctica. Ann NY Acad Sci 1223: 82–107. 21449967 10.1111/j.1749-6632.2010.05926.x

[25] Richardson MG (1979) The ecology and reproduction of the brooding Antarctic bivalve *Lissarca miliaris* . Br Antarct Surv Bull 49: 91–151.

[26] Reed AJ, Thatje S, Linse K (2012) An unusual hermaphrodite reproductive trait in the Antarctic brooding bivalve *Lissarca miliaris* (Philobryidae) from the Scotia Sea, Southern Ocean. Polar Biol DOI: 10.1007/s00300-012-1233-0.

[27] Arntz WA, Brey T (2003) Expedition ANTARKTIS XIX/5 (LAMPOS) of RV “Polarstern” in 2002. Rep Polar Mar Res 462: 1–122.

[28] Higgs ND, Reed AJ, Hooke R, Honey DJ, Heilmayer O, et al (2009) Growth and reproduction in the Antarctic brooding bivalve *Adacnarca nitens* (Philobryidae) from the Ross Sea. Mar Biol 156: 1073–1081.

[29] Brey T, Hain S (1992) Growth, reproduction and production of *Lissarca notorcadensis* (Bivalvia: Philobryidae) in the Weddell Sea, Antarctica. Mar Ecol Prog Ser 82: 219–226.

[30] Appeldoorn RS (1982) Variation in the growth rate of *Mya arenaria* and its relationship to the environment as analysed through principal components analysis and the ω parameter of the von Bertalanffy equation. Fish Bull 81: 75–84.

[31] Clarke A, Holmes LJ, White MG (1988) The annual cycle of temperature, chlorophyll and nutrients at Signy Island, South Orkney Islands, 1969–82. Br Antarct Surv Bull 80: 65–86.

[32] Clarke A, Leakey JG (1996) The seasonal cycle of phytoplankton, macronutrients, and the microbial community in a near shore Antarctic marine ecosystem. Limnol Oceanogr 41: 1281–1294.

[33] Huber M (2010) *Lissarca miliaris* (Philippi, 1845). The SCAR-MarBIN Register of Antarctic Marine Species (RAMS). Available: http://www.scarmarbin.be/rams.php?p=taxdetails&id=197240. Accessed 06 September 2012.

[34] Brey T (1999) Growth performance and mortality in aquatic benthic invertebrates. Adv Mar Biol 35: 153–223.

[35] Heilmayer O, Brey T, Pörtner H-O (2004) Growth efficiency and temperature in scallops: a comparative analysis of species adapted to different temperatures. Funct Ecol 18: 641–647.

[36] de Villiers S, Nelson BK, Chivas AR (1995) Biological controls on coral Sr/Ca and δ^18^O reconstructions of sea surface temperatures. Science 269: 1247–1249. 17732111 10.1126/science.269.5228.1247

[37] Rosenheim BE, Swart PK, Thorrold SR, Willenz P, Berry L, et al (2004) High resolution Sr/Ca records in sclerosponges calibrated to temperature in situ. Geology 32: 145–148.

[38] Watson EB (2004) A conceptual model for near-surface kinetic controls on the trace-element and stable isotope composition of abiogenic calcite crystals. Geochim Cosmochim Acta 68: 1473–1488.

[39] Sosdian S, Gentry DK, Lear CH, Grossman EL, Hicks D, et al (2006) Strontium to calcium ratios in the marine gastropod *Conus ermineus*: Growth rate effects and temperature calibration. Geochem Geophys Geosyst 7: 1–17.

[40] Webb SC, Korrûbel JL (1994) Shell weakening in marine mytilids attributable to blue-green alga *Mastigocoleus* sp. *(Nostochopsidaceae)* . J Shellfish Res 13: 11–17.

[41] Kaehler S (1999) Incidence and distribution of phototrophic shell-degrading endoliths of the brown mussel *Perna perna* . Mar Biol 135: 505–514.

[42] Cerrano C, Bavestrello G, Calcinai B, Cattaneo-Vietti R, Chiantore M, et al (2001) Bioerrosive processes in Antarctic seas. Polar Biol 24: 790–792.

[43] Nolan CP (1991) Size, shape and shell morphology in the Antarctic limpet *Nacella concinna* at Signy Island, South Orkney Islands. J Moll Stud 57: 225–238.

[44] Geller JB (1990) Reproductive responses to shell damage by the gastropod *Nucella emarginata* (Deshayes). J Exp Mar Biol Ecol 136: 77–87.

[45] Kaehler S, McQuaid CD (1999) Lethal and sub-lethal effects of phototrophic endoliths attacking the shell of the inter-tidal mussel *Perna perna* . Mar Biol 135: 497–503.

[46] Goodsell JG, Eversole AG (1992) Prodissconch I and II length in *Mercenaria* taxa. Nautilus 106: 119–122.

[47] Taskinen J, Saarinen M (1999) Increased parasite abundance associated with reproductive maturity of the clam *Anodonta piscinalis* . J Parasitol 85: 588–591. 10386464

[48] Halpern BS, Walbridge S, Selkoe KA, Kappel CV, Micheli F, et al (2008) A global map of human impact on marine ecosystems. Science 319: 948–952. 18276889 10.1126/science.1149345

