# Supplementary material for: Shifting Baselines in Antarctic Ecosystems; Ecophysiological Response to Warming in Lissarca miliaris at Signy Island, Antarctica
Source: PLoS One. 2012 Dec 28;7(12):e53477. doi: 10.1371/journal.pone.0053477 (PMC3532442; doi:10.1371/journal.pone.0053477)

Figure S1.
Mean annual temperature from 1947-1995 from the British Signy Island research station (dotted line) and Argentine Orcadas, Laurie Island research station (solid line). Signy Island temperature data collection stopped in 1995.

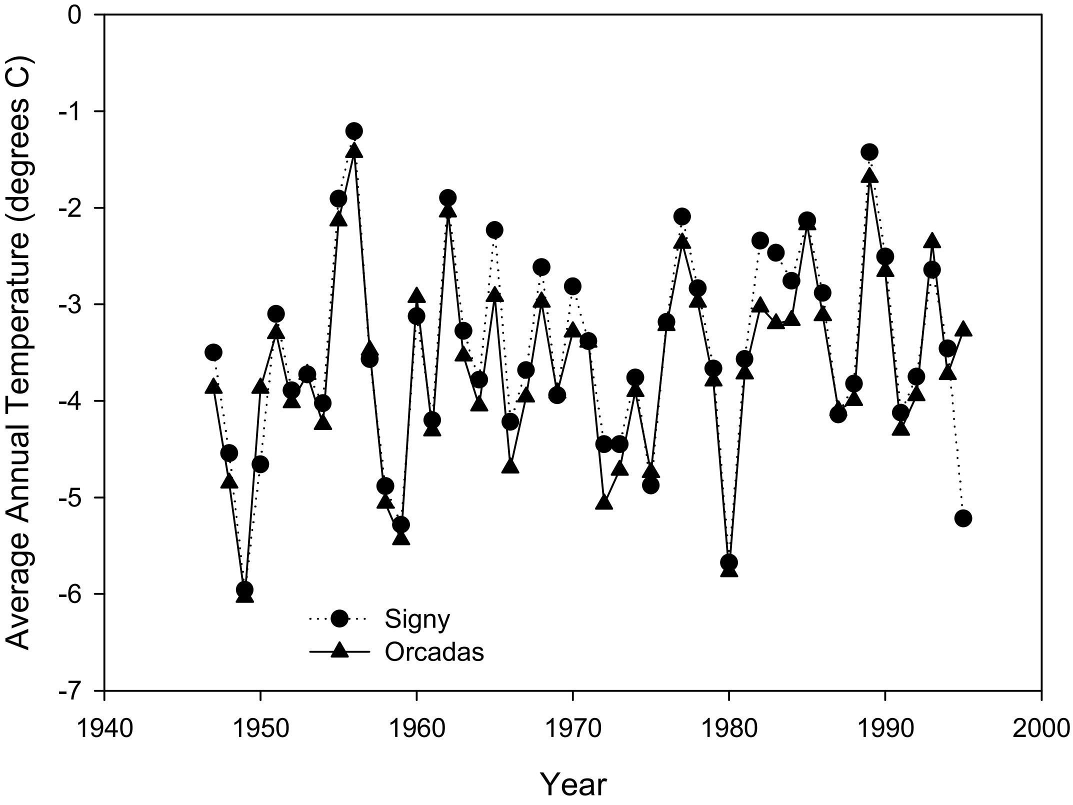

Supplement: Figure S1 — Mean annual temperature from 1947–1995 from the British Signy Island base (dotted line) and Argentine Orcadas, Laurie Island Base (solid line). Signy Island temperature data collection stopped in 1995. (PDF) [file pone.0053477.s001.pdf]
