# Supplementary material for: Shifting Baselines in Antarctic Ecosystems; Ecophysiological Response to Warming in Lissarca miliaris at Signy Island, Antarctica
Source: PLoS One. 2012 Dec 28;7(12):e53477. doi: 10.1371/journal.pone.0053477 (PMC3532442; doi:10.1371/journal.pone.0053477)

Figure S2. Adult *Lissarca miliaris* from Shallow Bay, Signy Island collected in 2012. Endolithic algae can still be seen in green, covering the shells shortly after fixation. Scale bars = 2 mm

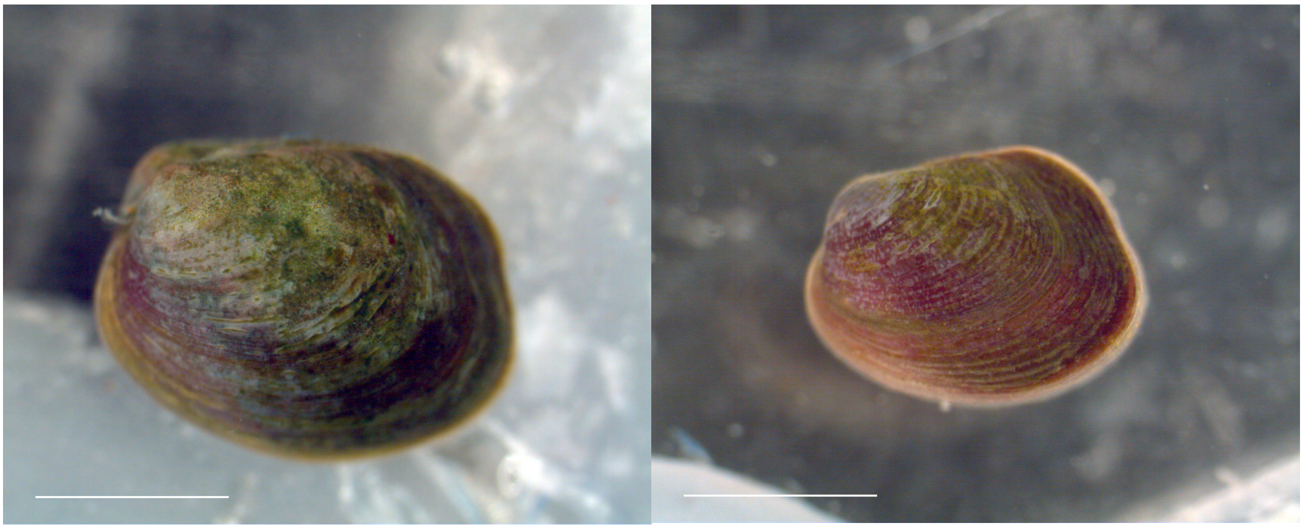

Supplement: Figure S2 — Adult Lissarca miliaris from Shallow Bay, Signy Island collected in 2012. Endolithic algae can still be seen in green, covering the shells shortly after fixation. Scale bars = 2 mm. (PDF) [file pone.0053477.s002.pdf]
