# Supplementary material for: Shifting Baselines in Antarctic Ecosystems; Ecophysiological Response to Warming in Lissarca miliaris at Signy Island, Antarctica
Source: PLoS One. 2012 Dec 28;7(12):e53477. doi: 10.1371/journal.pone.0053477 (PMC3532442; doi:10.1371/journal.pone.0053477)
Supplement: Table S1 — Parameters of the von Bertalanffy growth function and calculated overall growth performance (OGP) for Lissarca miliaris at Shallow Bay, Signy Island. (PDF) [file pone.0053477.s003.pdf]

Table S1. Parameters of the von Bertalanffy growth function and calculated overall growth performance (OGP) for *Lissarca miliaris* at Shallow Bay, Signy Island.

**Year**	**S_∞ (mm)**	***K***	**t_0**	**OGP**	**n**	**Ref**
1972	8.61	0.130	0.821	0.049	?	Richardson 1979
1976	8.87	0.127	0.212	0.052	236	this study
2002	6.39	0.208	0.065	0.124	439	this study
2011	5.88	0.290	0.232	0.232	66	this study
